# New Iboga-Type Indole Alkaloids from *Tabernaemontana divaricata*

**DOI:** 10.1007/s13659-019-00226-z

**Published:** 2019-11-14

**Authors:** Xiang-Mei Li, Xian-Jun Jiang, Guo-Zhu Wei, Li-Hua Ren, Li-Xia Wang, Xue-Lian Cheng, Fei Wang

**Affiliations:** 1 BioBioPha Co., Ltd., Kunming, 650201 People’s Republic of China; 2 Reference Substance Branch, National Engineering Research Center for Modernization of Traditional Chinese Medicine, Kunming, 650201 People’s Republic of China; 3 Department of Pharmacy, The People’s Hospital of Fengjie County, Chongqing, 404600 People’s Republic of China

**Keywords:** *Tabernaemontana divaricata*, Iboga-type indole alkaloid, Single crystal X-ray diffraction

## Abstract

**Abstract:**

Two hitherto unknown iboga-type indole alkaloids, namely (3*R*)-7,19-di-*epi*-3-methoxytabernoxidine (**1**) and (3*R*,19*R*)-19-hydroxy-3-(2-oxopropyl)voacangine (**2**), together with eight known alkaloids (**3**–**10**), were isolated from the twigs and leaves of *Tabernaemontana divaricata*. Their structures were established on the basis of spectroscopic data interpretation, single crystal X-ray diffraction analysis and circular dichroism spectrum.

**Graphic Abstract:**

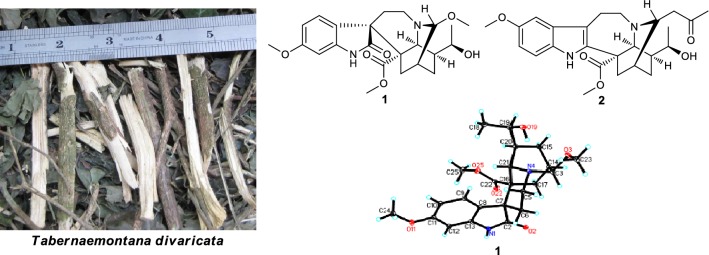

**Electronic supplementary material:**

The online version of this article (10.1007/s13659-019-00226-z) contains supplementary material, which is available to authorized users.

## Introduction

**Fig. 1:**
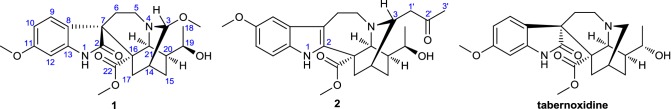
The structures of compounds **1**, **2** and tabernoxidine

The genus *Tabernaemontana* (Apocynaceae family), comprising 120 species, is a rich source of monoterpenoid indole alkaloids with diverse skeletons, particularly iboga-type alkaloids [1–3]. *Tabernaemontana divaricata*, distributed in Yunnan and Guangxi Provinces of China [4], is widely used as folk medicine for the treatment of sore throat and hypertension [5], and a series of monoterpenoid indole and bisindole alkaloids were reported [6, 7]. As part of a BioBioPha (https://www.chemlib.cn) objective to assemble a large-scale natural product library valuable in the discovery of new drug leads from nature, the phytochemical investigation on the twigs and leaves of *T*. *divaricata* resulted in the isolation of two new iboga-type indole alkaloids, namely (3*R*)-7,19-di-*epi*-3-methoxytabernoxidine (**1**) and (3*R*,19*R*)-19-hydroxy-3-(2-oxopropyl)voacangine (**2**) (Fig. 1), together with eight known indole alkaloids: 19,20-(*E*)-vallesamine (**3**) [8, 9], ibogaine (**4**) [10], conophylline (**5**) [11], taberdivarine H (**6**) [12], conodurine (**7**) [13], hecubine (**8**) [14], voafinidine (**9**) [15], and tabernanthine (**10**) [10, 16]. In this paper, we report the isolation and structure elucidation of new alkaloids **1** and **2**.

## Results and Discussion

**Fig. 2:**
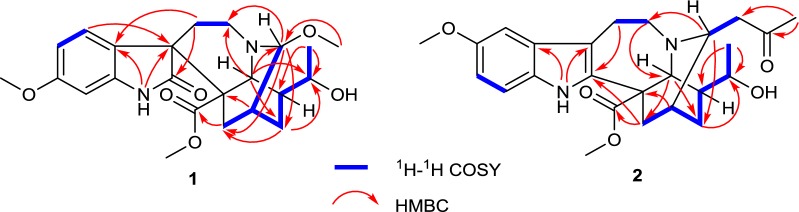
Key ^1^H–^1^H COSY (  ) and HMBC (  ) correlations of **1** and **2**

**Fig. 3:**
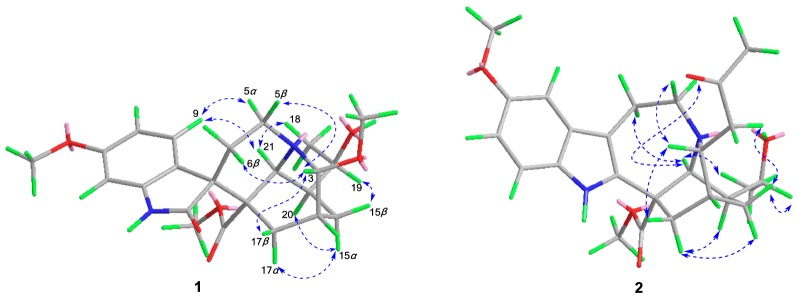
Key ROESY (  ) correlations of **1** and **2**

**Fig. 4:**
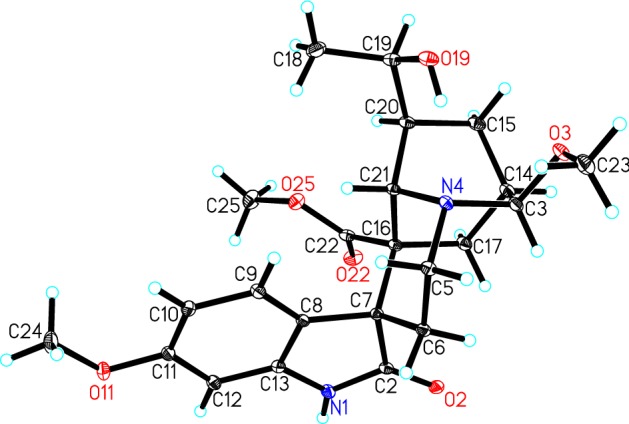
X-ray crystallographic structure of compound **1**

Compound **1**, obtained as colorless block crystals from MeOH, possessed a molecular formula of C_23_H_30_N_2_O_6_, as evidenced by HR-ESI-MS (pos.) at *m/z* 453.1997 [M + Na]^+^ (calcd. for C_23_H_30_N_2_O_6_Na, 453.1996), requiring 10 degrees of unsaturation. The ^1^H NMR spectrum in chloroform-*d* (Table 1) displayed one NH proton at *δ*_H_ 7.90 (1H, s), one 1,2,4-trisubstituted benzene ring at *δ*_H_ 7.63 (1H, d, *J* = 8.5 Hz), 6.54 (1H, dd, *J* = 8.5, 2.6 Hz), and 6.43 (1H, d, *J* = 2.6 Hz), two oxygenated methines at *δ*_H_ 4.18 (1H, d, *J* = 3.3 Hz) and 3.95 (1H, m), one secondary methyl at *δ*_H_ 1.24 (3H, d, *J* = 6.4 Hz), and three methoxy groups at *δ*_H_ 3.78, 3.33, and 3.19 (each 3H, s). The ^13^C NMR spectrum (Table 1) displayed a total of 23 carbon resonances, including two overlapping signals (C-5 and C-16). Combined with the DEPT spectrum, different types of carbons were distinguished to be four methyls, four methylenes, eight methines and seven quaternary carbons (including two carbonyls at *δ*_C_ 179.2 and 174.3). The HSQC and ^1^H-^1^H COSY spectra (Fig. 2) revealed the connections of C-3/C-14 and C-17/C-14/C-15/C-20/C-19/C-18, which were confirmed by the HMBC correlations (Fig. 2) from H-3 to C-15 and C-17, H-15 to C-17 and C-19, and Me-18 to C-19 and C-20. The above NMR analysis indicated the planar structure of **1** was remarkably similar to that of tabernoxidine (Fig. 1) [17], an iboga-type spiro oxindole alkaloid whose absolute stereochemistry has been established by single crystal X-ray diffraction. The prominent difference between them was the absence of resonance for a methylene (C-3) and the presence of resonances for a downfield oxygenated methine and a methoxy. The oxygenated methine was assigned to C-3 based on the HMBC correlations from H-21 to C-3, and from H-3 to C-5, C-15, and C-17. And the methoxy was positioned at C-3 by the HMBC correlation between the methoxy protons and C-3. In the ROESY spectrum (Fig. 3), the correlations between H-9 and H-21/H-5*α* revealed R* configuration of the spiro carbon atom at C-7, and the correlations between H-3 and H-5*β*/H-6*β/*H-17*β* indicated R* configuration of C-3. The above deduction was confirmed by single crystal X-ray diffraction using Mo K*α* radiation (Fig. 4). Since the absolute stereochemistry of iboga-type indole alkaloids from Apocynaceae had been determined previously by single crystal X-ray diffraction using Cu K*α* radiation or electronic circular dichroism (ECD) analysis [1–3, 17], biogenetically, the absolute configuration of **1** could be deduced as 3*R*,7*R*,14*R*,16*R*,19*R*,20*S*,21*S*. Therefore, the structure of **1** was established and named as (3*R*)-7,19-di-*epi*-3-methoxytabernoxidine, as shown in Fig. 1.

**Table 1:** ^1^H and ^13^C NMR spectroscopic data of compounds **1** and **2** in CDCl_3_ (*δ*_H_ 7.26, *δ*_C_ 77.0 ppm)

No.	**1**		**2**	
	*δ*_H_	*δ*_C_	*δ*_H_	*δ*_C_
1	7.90 (s)		7.70 (s)	
2		179.2 (s)		136.3 (s)
3	4.18 (d, 3.3)	91.5 (d)	3.38 (dd, 8.6, 4.0)	54.2 (d)
5	3.46 (ddd, 14.6, 14.2, 3.5, H_*α*_) 3.05 (br dd, 14.6, 5.1, H_*β*_)	48.7 (t)	3.34 (m, H_*α*_) 3.15 (m, H_*β*_)	50.4 (t)
6	2.39 (td, 14.2, 5.1, H_*β*_) 1.28 (br dd, 14.2, 3.5, H_*α*_)	26.2 (t)	3.14 (m, H_*β*_) 3.04 (m, H_*α*_)	21.7 (t)
7		50.6 (s)		109.5 (s)
8		123.6 (s)		128.9 (s)
9	7.63 (d, 8.5)	126.9 (d)	6.89 (d, 2.4)	100.7 (d)
10	6.54 (dd, 8.5, 2.6)	106.5 (d)		154.1 (s)
11		160.0 (s)	6.83 (dd, 8.7, 2.4)	112.4 (d)
12	6.43 (d, 2.6)	97.0 (d)	7.15 (d, 8.7)	111.2 (d)
13		140.7 (s)		130.6 (s)
14	2.16 (m)	30.1 (d)	1.86 (br s)	30.5 (d)
15	1.64 (m, H_*β*_) 1.32 (m, H_*α*_)	21.4 (t)	1.90 (m, H_*β*_) 1.63 (m, H_*α*_)	24.0 (t)
16		48.7 (s)		53.7 (s)
17	3.18 (m, H_*β*_) 1.96 (dd, 15.8, 2.9, H_*α*_)	27.2 (t)	2.63 (dd, 13.8, 1.8, H_*α*_) 2.09 (ddd, 13.8, 4.0, 3.0, H_*β*_)	37.7 (t)
18	1.24 (d, 6.4)	21.9 (q)	1.28 (d, 6.5)	22.2 (q)
19	3.95 (m)	70.2 (d)	3.89 (qd, 6.5, 2.0)	71.0 (d)
20	1.84 (m)	39.2 (d)	1.35 (m)	39.7 (d)
21	3.95 (br s)	50.4 (d)	4.10 (s)	55.2 (d)
22		174.3 (s)		174.7 (s)
3-OMe	3.33 (s)	53.7 (q)		
10-OMe			3.84 (s)	56.0 (q)
11-OMe	3.78 (s)	55.4 (q)		
22-OMe	3.19 (s)	51.4 (q)	3.72 (s)	52.9 (q)
1′			2.77 (dd, 17.3, 8.6) 2.69 (dd, 17.3, 4.0)	45.1 (t)
2′				207.6 (s)
3′			2.13 (s)	31.0 (q)

Compound **2**, white amorphous powder, gave a molecular formula of C_25_H_32_N_2_O_5_ as determined by positive HRESIMS at *m/z* 441.2384 [M + H]^+^ (calcd. for C_25_H_33_N_2_O_5_, 441.2384) with 11 degrees of unsaturation. The ^1^H and ^13^C NMR spectra (Table 1) showed that **2** had a structure similar to that of 3-(2-oxopropyl)voacangine [18], except for the presence of a 1-hydroxyethyl group [*δ*_H_ 3.89 (1H, qd, *J* = 6.5, 2.0 Hz)/*δ*_C_ 71.0 (d) and *δ*_H_ 1.28 (3H, d, *J* = 6.5 Hz)/*δ*_C_ 22.2 (q)] in place of an ethyl side chain at C-20, which was confirmed by the HMBC correlations (Fig. 2) from Me-18 to C-19 and C-20. The ROESY correlations (Fig. 3) of H-21 ↔ H-5*α*/H-6*α*, H-17*α* ↔ H-20/H-15*α* suggested that these protons were *α*-oriented, and the correlations of H-3 ↔ H-17*β*, H-1′ ↔ H-15*β* revealed the stereochemistry of C-3, which indicated that the configurations of C-3, C-14, C-16, C-20 and C-21 were identical to those in 3-(2-oxopropyl)voacangine. Comparison of the ^13^C NMR chemical shifts of C-15 and C-21 as well as the ^1^H NMR chemical shift of H-19 with those of 19*R*/*S*-hydroxyiboga alkaloids indicated a 19*R* configuration [19, 20]. Additionally, the Cotton effects in the CD spectrum (See S14, Supplementary Material) of **2** were in good agreement with those for voacangine [18], so the absolute configuration of **2** was assigned as 3*R*,14*R*,16*S*,19*R*,20*S*,21*S*. Hence, the structure of **2** was established and named as (3*R*,19*R*)-19-hydroxy-3-(2-oxopropyl)voacangine.

## Experimental Section

### General Experimental Procedures

X-ray data were collected using a Bruker APEX DUO diffractometer with graphite-monochromated Mo K*α* radiation. Optical rotations were measured on a Rudolph Autopol VI automatic polarimeter. UV data were obtained from HPLC online analysis. IR spectra (KBr) were obtained on a Thermo Nicolet iS10 FT-IR spectrometer. NMR spectra were carried out on a Bruker Avance III 600 or DRX-500 spectrometer with deuterated solvent signals as internal standards. ESIMS and HRESIMS were measured using an Agilent G6230 time-of-flight mass spectrometer. Preparative HPLC separation was performed using an Agilent 1260 series HPLC system equipped with a Zorbax SB-C_18_ column (5 μm, 21.2 × 150 mm). Silica gel (200–300 mesh, Qingdao Marine Chemical Inc., China), Chromatorex C_18_ (40–75 μm, Fuji Silysia Chemical Ltd., Japan) and Sephadex LH-20 (GE Healthcare Bio-Sciences AB, Uppsala, Sweden) were used for column chromatography. Fractions were monitored and analyzed using TLC, in combination with Agilent 1200 series HPLC system equipped by an Extend-C_18_ column (5 μm, 4.6 × 150 mm).

### Plant Material

The twigs and leaves of *Tabernaemontana divaricata* were collected from Menglun town, Xishuangbanna Dai Autonomous Prefecture in Yunnan Province, China, in January 2014 and identified by Mr. Yu Chen of Kunming Institute of Botany, Chinese Academy of Sciences. A voucher specimen (No. BBP0671) was deposited at BioBioPha Co., Ltd.

### Extraction and Isolation

The twigs and leaves of *T*. *divaricata* were extracted with 95% aqueous EtOH at room temperature, and the solvent was removed under reduced pressure to give crude extract (*ca.* 600 g), then dissolved in 2% HCl and filtered. The filtration was basified using 10% ammonia-water to pH 9−10 and then partitioned with EtOAc to give a total alkaloidal extract (*ca*. 100 g), which was fractionated by silica gel CC successively eluted with CHCl_3_/MeOH (200:1 → 10:1 gradient) to give six fractions A –E, respectively. Fraction B was separated by silica gel CC (CHCl_3_/MeOH, 100:1 → 80:1) into fractions B_1_ –B_4_. Fraction B_1_ was purified by silica gel CC using petroleum ether (PE)/acetone (4:1) and Sephadex LH-20 (CHCl_3_/MeOH, 1:1) to obtain **7** (50 mg). Fraction B_2_ was separated by silica gel CC (CHCl_3_/MeOH, 100:1) to give **8** (18 mg) and **9** (4 mg). Fraction C was separated by silica gel (CHCl_3_/MeOH, 100:1 → 60:1) into fractions C_1_–C_3_. Fraction C_1_ was separated by Sephadex LH-20 (CHCl_3_/MeOH, 1:1) to obtain compound **5** (17 mg). Fraction C_2_ was separated by silica gel (CHCl_3_/MeOH, 60:1), prep-HPLC (MeOH/H_2_O; 60%) and Sephadex LH-20 (CHCl_3_/MeOH, 1:1) to obtain compounds **1** (46 mg), **4** (3 mg) and **10** (102 mg). Fraction D was separated by silica gel CC (CHCl_3_/MeOH, 60:1 → 30:1) into fractions D_1_–D_4_. Fraction D_2_ was further purified by silica gel (CHCl_3_/MeOH, 80:1) and prep-HPLC (MeOH/H_2_O; 80%) to give **2** (14 mg). Fraction E was subjected to MPLC (MeOH/H_2_O, 50%) and then by Sephadex LH-20 (MeOH) to give **3** (33 mg) and **6** (13 mg). The retention times (*t*_R_) of **1** and **2** on an analytical HPLC InertSustain C_**18**_ column (20% → 100% MeOH in H_2_O over 8.0 min followed by 100% MeOH to 13.0 min, 1.0 ml/min) were 4.60 and 6.00 min, respectively.

### (3*R*)-7,19-Di-*epi*-3-methoxytabernoxidine (1)

Colorless block crystals; [*α*]_D_^22^ + 25.9 (*c* 0.10, MeOH); UV (MeOH) *λ*_max_: 217, 261, 285 (sh) nm; IR (KBr) *ν*_max_: 3427, 2966, 2945, 2936, 2901, 2835, 1720, 1631, 1594, 1507, 1468, 1365, 1314, 1197, 1160, 1101, 829 cm^−1^; ^1^H and ^13^C NMR data: see Table 1; ESIMS (pos.): *m/z* 453 [M + Na]^+^; HRESIMS (pos.): *m/z* 453.1997 [M + Na]^+^ (calcd. for C_23_H_30_N_2_O_6_Na, 453.1996).

### Crystallographic Data for 1

C_23_H_30_N_2_O_6_·CH_4_O, *M* = 462.53, monoclinic, *a* = 10.9955(11) Å, *b* = 6.7753(7) Å, *c* = 15.2342(15) Å, *α* = 90.00°, *β* = 92.975(2)°, *γ* = 90.00°, *V* = 1133.4(2) Å^3^, *T* = 100(2) K, space group *P*21, *Z* = 2, *μ*(MoKα) = 0.100 mm^−1^, 12217 reflections measured, 6280 independent reflections (*R*_*int*_ = 0.0225). The final *R*_*1*_ values were 0.0339 (*I* > 2*σ*(*I*)). The final *wR*(*F*^2^) values were 0.0866 (*I* > 2*σ*(*I*)). The final *R*_*1*_ values were 0.0385 (all data). The final *wR*(*F*^2^) values were 0.0893 (all data). The goodness of fit on *F*^2^ was 1.043. Flack parameter = 0.3(3).

### (3*R*,19*R*)-19-Hydroxy-3-(2-oxopropyl)voacangine (2)

White amorphous powder; [*α*]_D_^22^ − 39.3 (*c* 0.10, MeOH); UV (MeOH) *λ*_max_: 223, 287 nm; IR (KBr) *ν*_max_: 3442, 2934, 2871, 1711, 1627, 1487, 1455, 1225, 1166, 1077, 1033, 828, 798 cm^−1^; ^1^H and ^13^C NMR data: see Table 1; ESIMS (pos.): *m/z* 441 [M + H]^+^; HRESIMS (pos.): *m/z* 441.2384 [M + H]^+^ (calcd. for C_25_H_33_N_2_O_5_, 441.2384).

## Electronic supplementary material

Below is the link to the electronic supplementary material.

Supplementary file1 (DOCX 786 kb)
