# Supplementary material for: New Iboga-Type Indole Alkaloids from Tabernaemontana divaricata
Source: Nat Prod Bioprospect. 2019 Nov 14;9(6):425–9. doi: 10.1007/s13659-019-00226-z (PMC6872691; doi:10.1007/s13659-019-00226-z)

**Electronic Supplementary Material**

**New Iboga-type Indole Alkaloids from** [***Tabernaemontana divaricata***](http://foc.iplant.cn/content.aspx?TaxonId=210002194)

**Xiang-Mei Li ^1,2^ • Xian-Jun Jiang ^1,2^ • Guo-Zhu Wei ^1,2^ • Li-Hua Ren ^1,2^ • Li-Xia Wang ^1,2^ • Xue-Lian Cheng ^1,3^ • Fei Wang ^1,2^**

^1^ BioBioPha Co., Ltd., Kunming 650201, People’s Republic of China

^2^ Reference Substance Branch, National Engineering Research Center for Modernization of Traditional Chinese Medicine, Kunming 650201, People’s Republic of China

^3^ Department of Pharmacy, the People’s Hospital of Fengjie County, Chongqing 404600, People’s Republic of China

*To whom correspondence should be addressed.

E-mail: f.wang@mail.biobiopha.com

The structures of compounds **1** and **2**

**Content list**

**S1.** ^1^H NMR spectrum (600 MHz, CDCl_3_) of (3*R*)-7,19-di-*epi*-3-methoxytabernoxidine (**1**).

**S2.** ^13^C NMR spectrum (125 MHz, CDCl_3_) of (3*R*)-7,19-di-*epi*-3-methoxytabernoxidine (**1**).

**S3.** **^1^**H-^1^H COSY spectrum (600 MHz, CDCl_3_) of (3*R*)-7,19-di-*epi*-3-methoxytabernoxidine (**1**).

**S4.** HSQC spectrum (600 MHz, CDCl_3_) of (3*R*)-7,19-di-*epi*-3-methoxytabernoxidine (**1**).

**S5.** HMBC spectrum (600 MHz, CDCl_3_) of (3*R*)-7,19-di-*epi*-3-methoxytabernoxidine (**1**).

**S6.** ROESY spectrum (600 MHz, CDCl_3_) of (3*R*)-7,19-di-*epi*-3-methoxytabernoxidine (**1**)

**S7.** X-Ray crystallographic data for (3*R*)-7,19-di-*epi*-3-methoxytabernoxidine (**1**).

**S8.** ^1^H NMR spectrum (500 MHz, CDCl_3_) of (3*R*,19*R*)-19-hydroxy-3-(2-oxopropyl)voacangine (**2**).

**S9.** ^13^C NMR spectrum (125 MHz, CDCl_3_) of (3*R*,19*R*)-19-hydroxy-3-(2-oxopropyl)voacangine (**2**).

**S10.** **^1^**H-^1^H COSY spectrum (500 MHz, CDCl_3_) of (3*R*,19*R*)-19-hydroxy-3-(2-oxopropyl)voacangine (**2**).

**S11.** HSQC spectrum (500 MHz, CDCl_3_) of (3*R*,19*R*)-19-hydroxy-3-(2-oxopropyl)voacangine (**2**).

**S12.** HMBC spectrum (500 MHz, CDCl_3_) of (3*R*,19*R*)-19-hydroxy-3-(2-oxopropyl)voacangine (**2**).

**S13.** ROESY spectrum (500 MHz, CDCl_3_) of (3*R*,19*R*)-19-hydroxy-3-(2-oxopropyl)voacangine (**2**).

**S14**. CD spectrum of (3*R*,19*R*)-19-hydroxy-3-(2-oxopropyl)voacangine (**2**).

**S1.** ^1^H NMR spectrum (600 MHz, CDCl_3_) of (3*R*)-7,19-di-*epi*-3-methoxytabernoxidine (**1**)


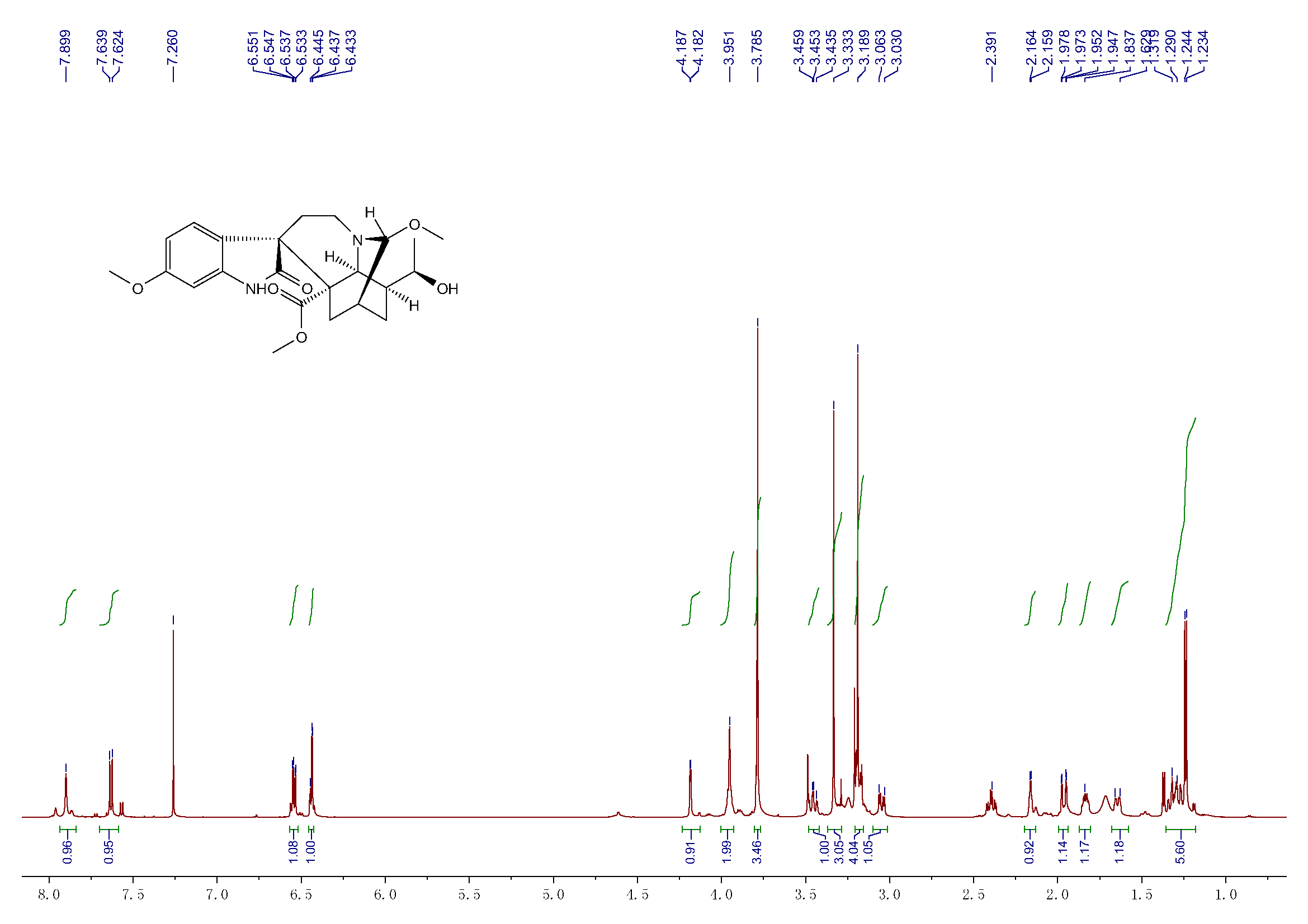


**S2.** ^13^C NMR spectrum (125 MHz, CDCl_3_) of (3*R*)-7,19-di-*epi*-3-methoxytabernoxidine (**1**)


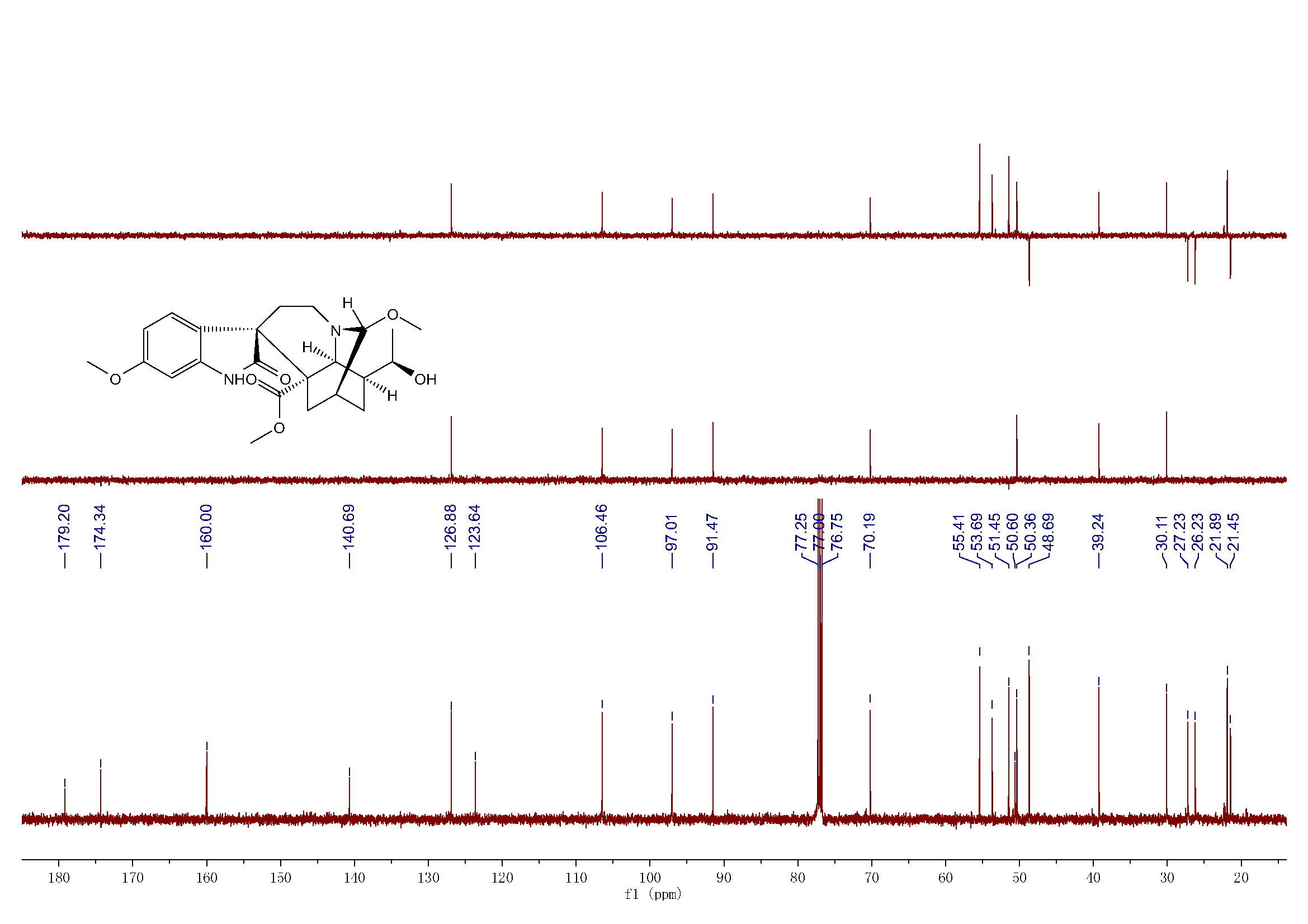


**S3.** **^1^**H-^1^H COSY spectrum (600 MHz, CDCl_3_) of (3*R*)-7,19-di-*epi*-3-methoxytabernoxidine (**1**)


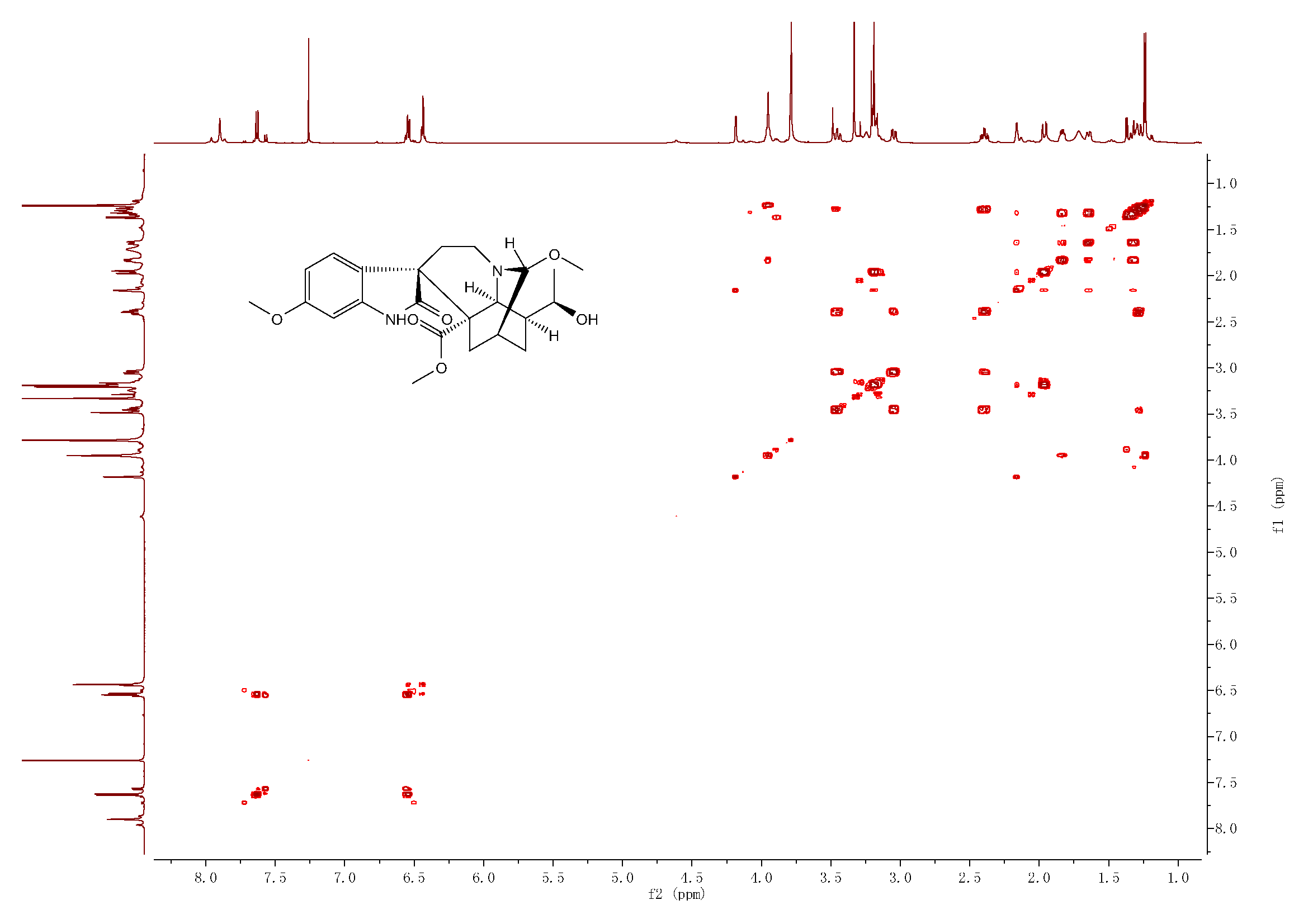


**S4.** HSQC spectrum (600 MHz, CDCl_3_) of (3*R*)-7,19-di-*epi*-3-methoxytabernoxidine (**1**)


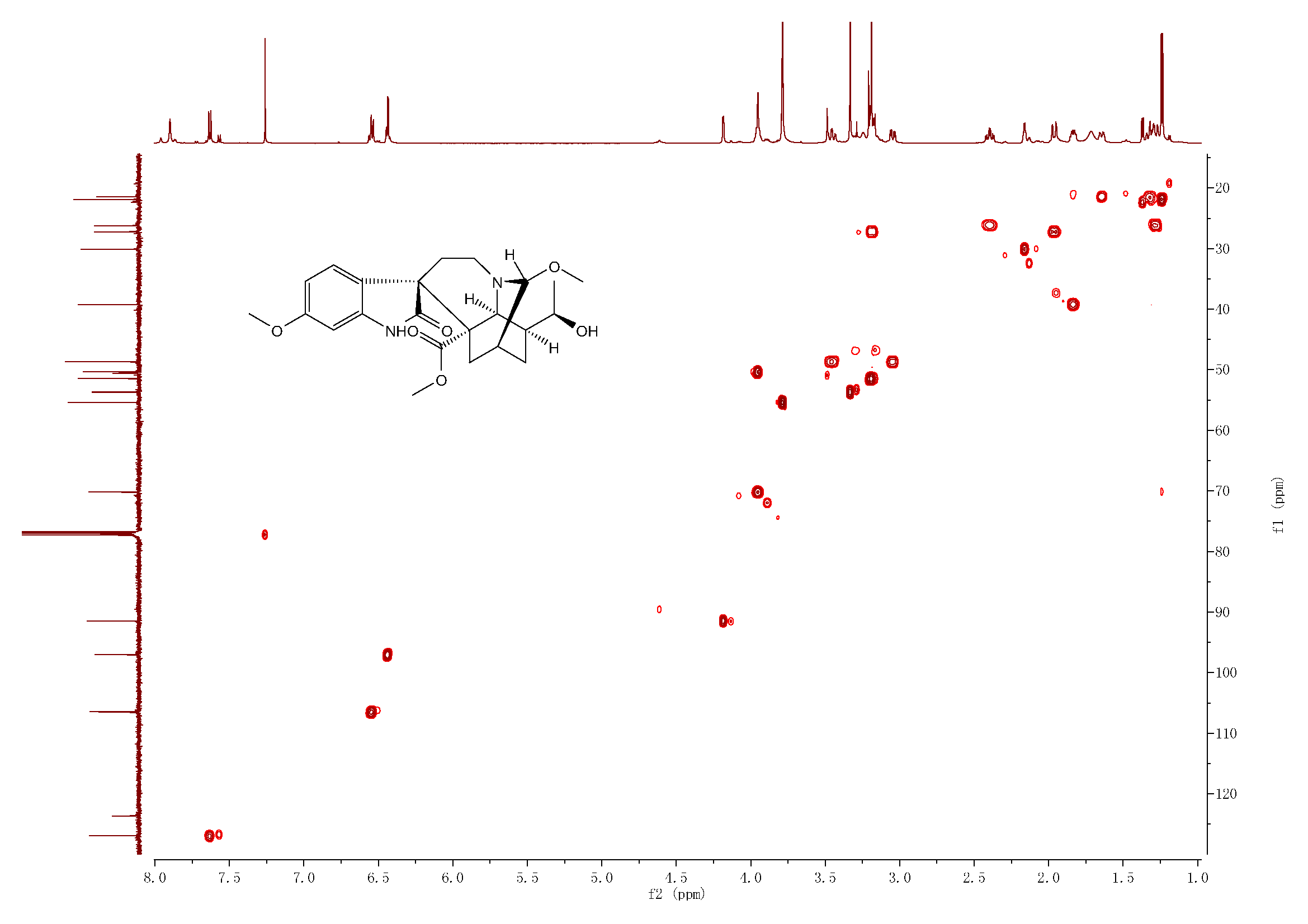


**S5.** HMBC spectrum (600 MHz, CDCl_3_) of (3*R*)-7,19-di-*epi*-3-methoxytabernoxidine (**1**)


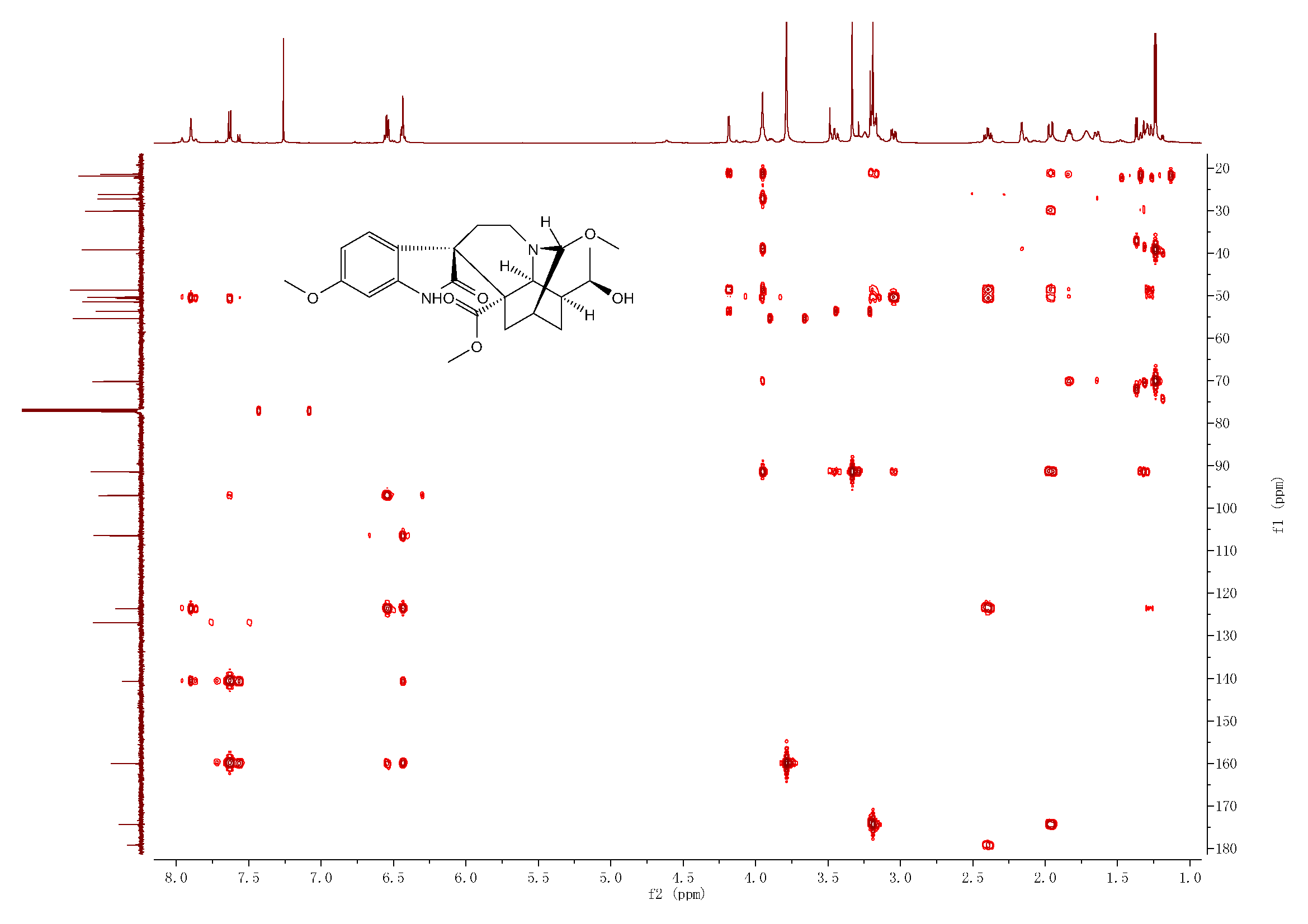


**S6.** ROESY spectrum (600 MHz, CDCl_3_) of (3*R*)-7,19-di-*epi*-3-methoxytabernoxidine (**1**)


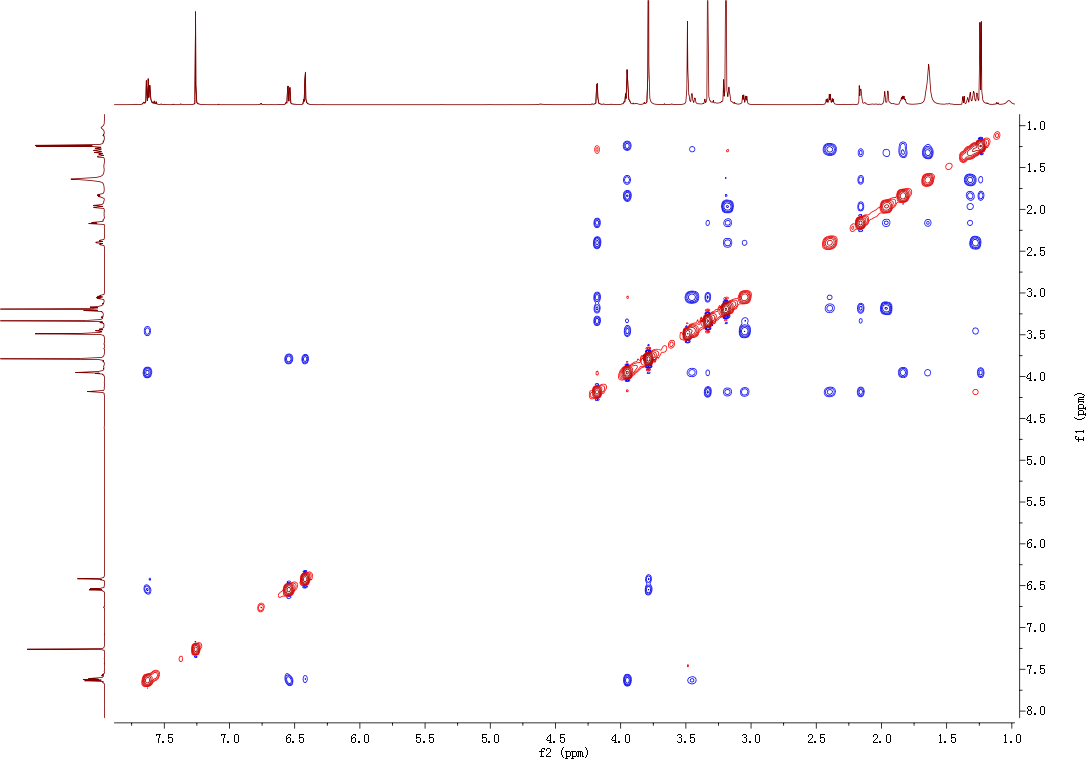


**S7. X-Ray crystallographic data for (3*R*)-7,19-di-*epi*-3-methoxytabernoxidine (1)**

Crystal data for **1**: C_23_H_30_N_2_O_6_•CH_4_O, *M* = 462.53, *a* = 10.9955(11) Å, *b* = 6.7753(7) Å, *c* = 15.2342(15) Å, *α* = 90°, *β* = 92.975(2)°, *γ* = 90°, *V* = 1133.4(2) Å^3^, *T* = 100(2) K, space group *P*21, *Z* = 2, *μ*(MoKα) = 0.100 mm^-1^, 12217 reflections measured, 6280 independent reflections (*R_int_* = 0.0225). The final *R_1_* values were 0.0339 (*I* > 2*σ*(*I*)). The final *wR*(*F*^2^) values were 0.0866 (*I* > 2*σ*(*I*)). The final *R_1_* values were 0.0385 (all data). The final *wR*(*F*^2^) values were 0.0893 (all data). The goodness of fit on *F*^2^ was 1.043. Flack parameter = 0.3(3).

View of the molecules in an asymmetric unit.

Displacement ellipsoids are drawn at the 30% probability level.

View of a molecule of **1** with the atom-labelling scheme.

Displacement ellipsoids are drawn at the 30% probability level.

View of the packing motif of **1**.

Hydrogen-bonds are shown as dashed lines.

**S8.** ^1^H NMR spectrum (500 MHz, CDCl_3_) of (3*R*,19*R*)-19-hydroxy-3-(2-oxopropyl)voacangine (**2**)


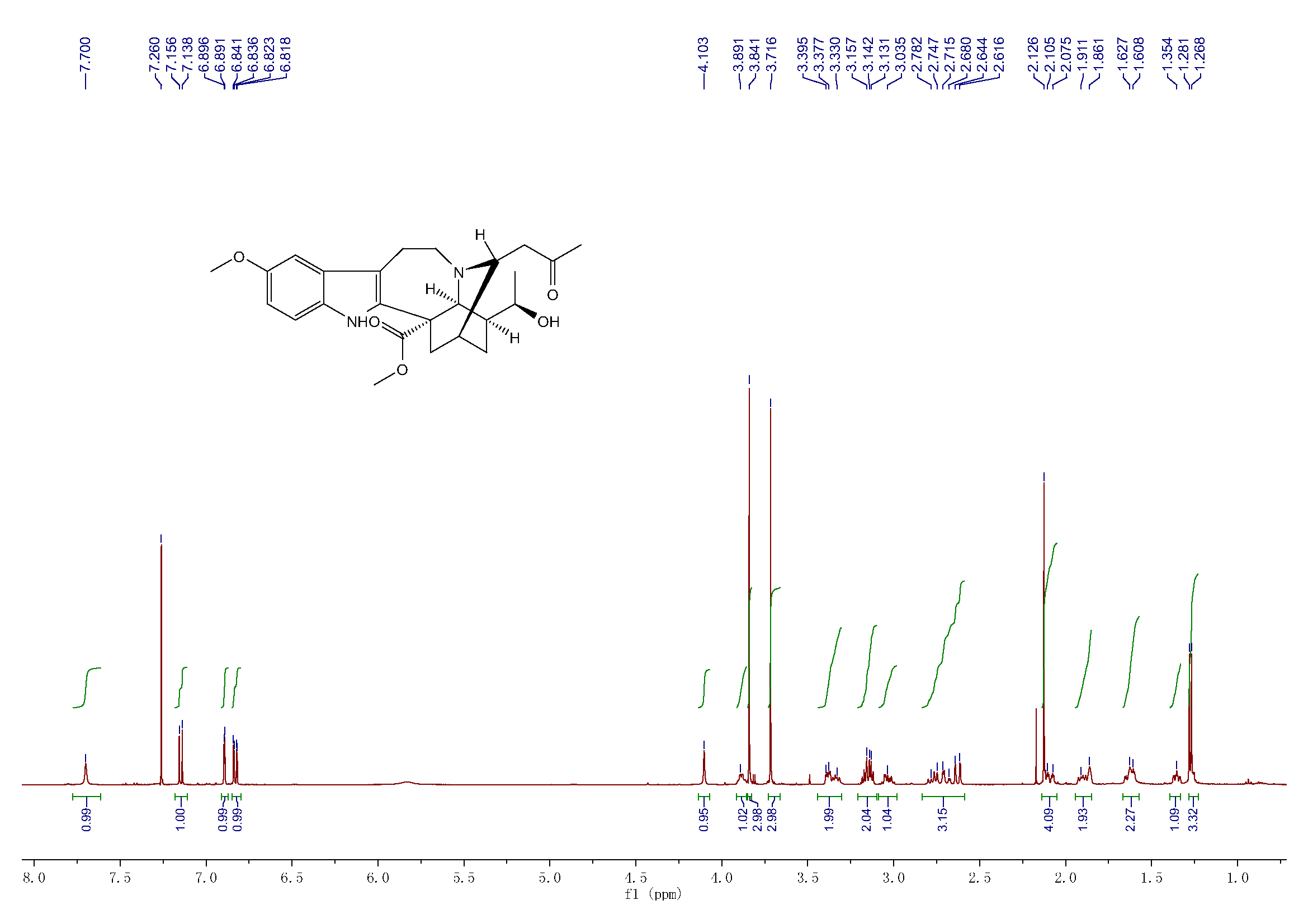


**S9.** ^13^C NMR spectrum (125 MHz, CDCl_3_) of (3*R*,19*R*)-19-hydroxy-3-(2-oxopropyl)voacangine (**2**)


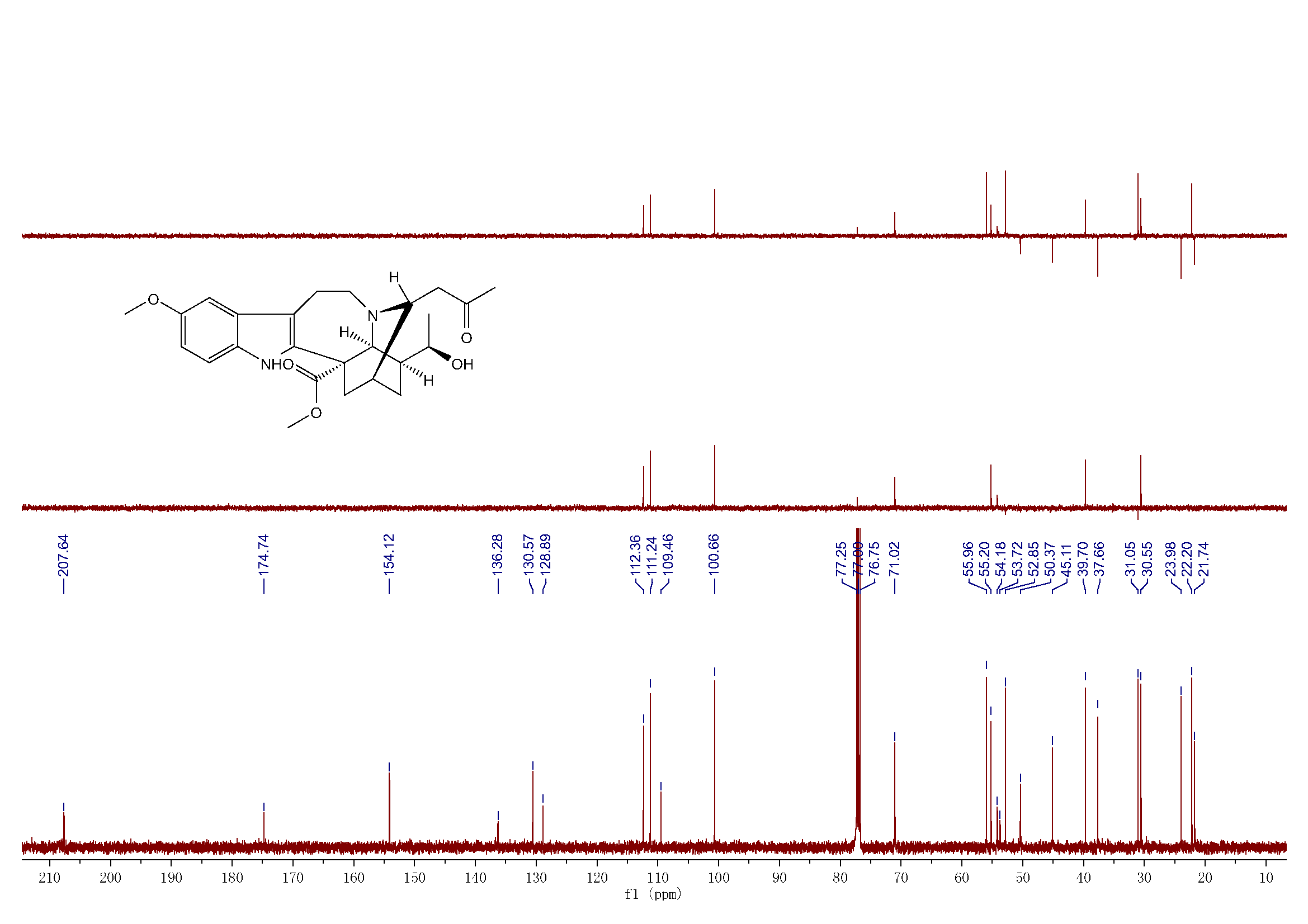


**S10.** ^1^H-^1^H COSY spectrum (500 MHz, CDCl_3_) of (3*R*,19*R*)-19-hydroxy-3-(2-oxopropyl)voacangine (**2**)


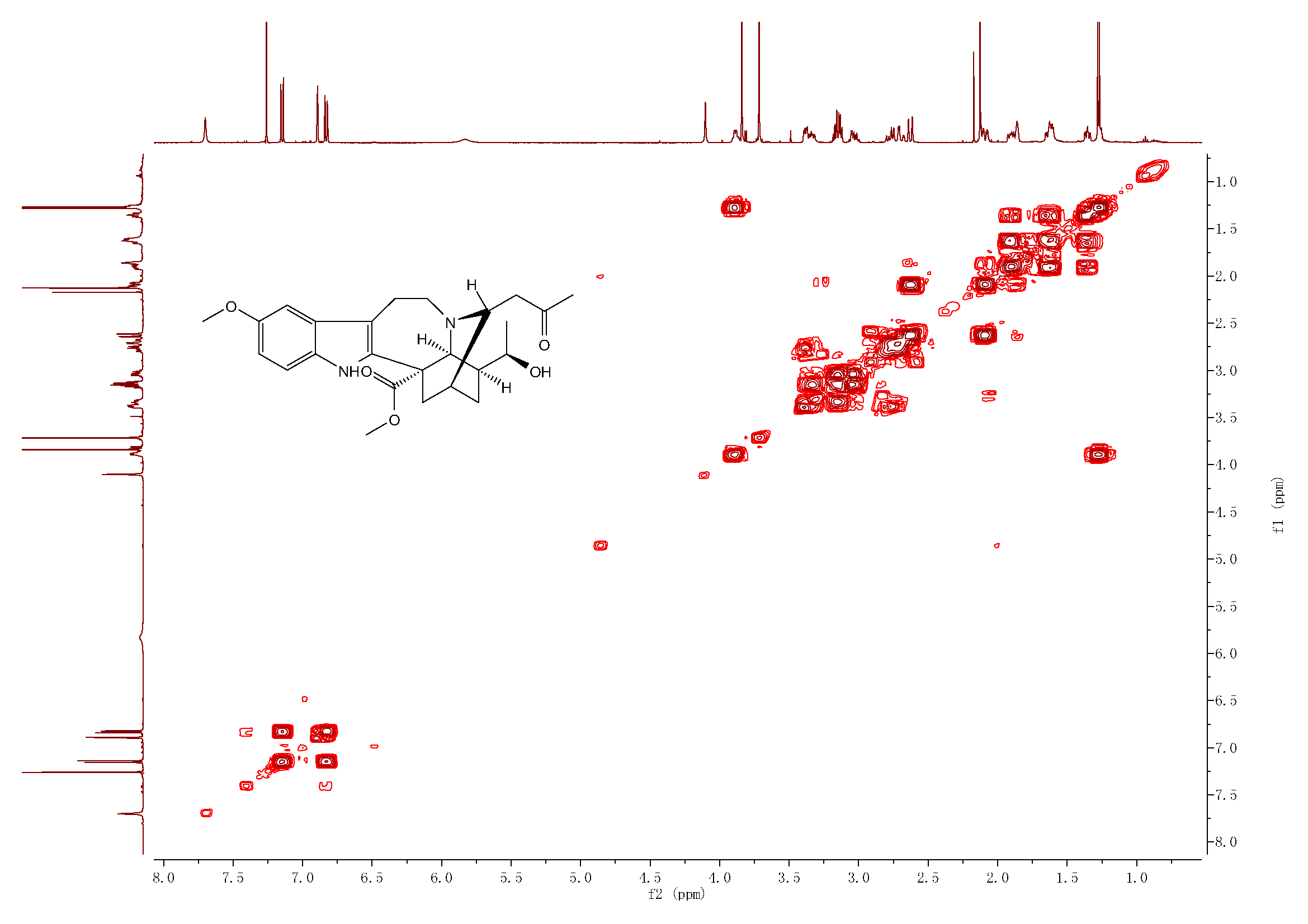


**S11.** HSQC spectrum (500 MHz, CDCl_3_) of (3*R*,19*R*)-19-hydroxy-3-(2-oxopropyl)voacangine (**2**)


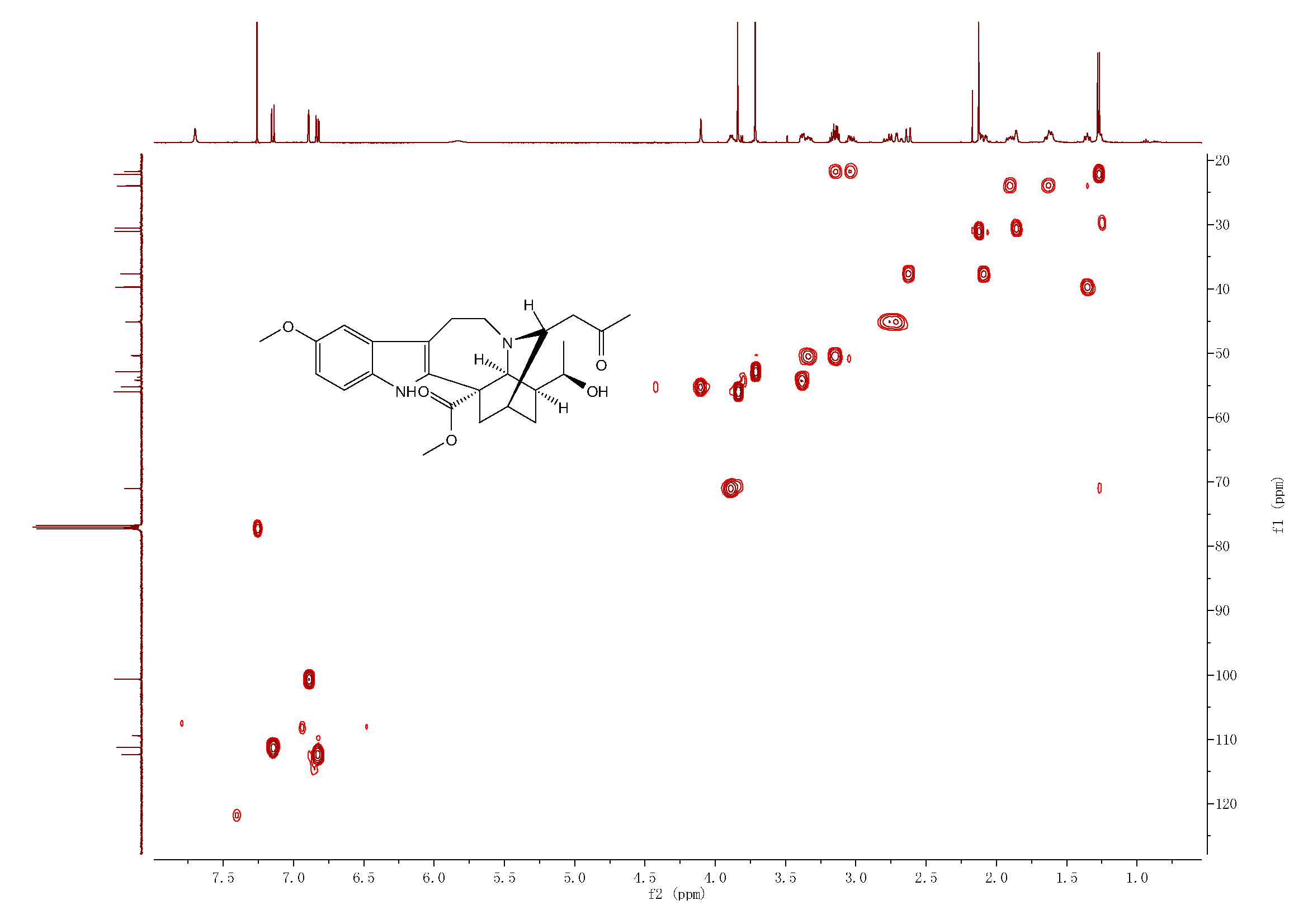


**S12.** HMBC spectrum (500 MHz, CDCl_3_) of (3*R*,19*R*)-19-hydroxy-3-(2-oxopropyl)voacangine (**2**)


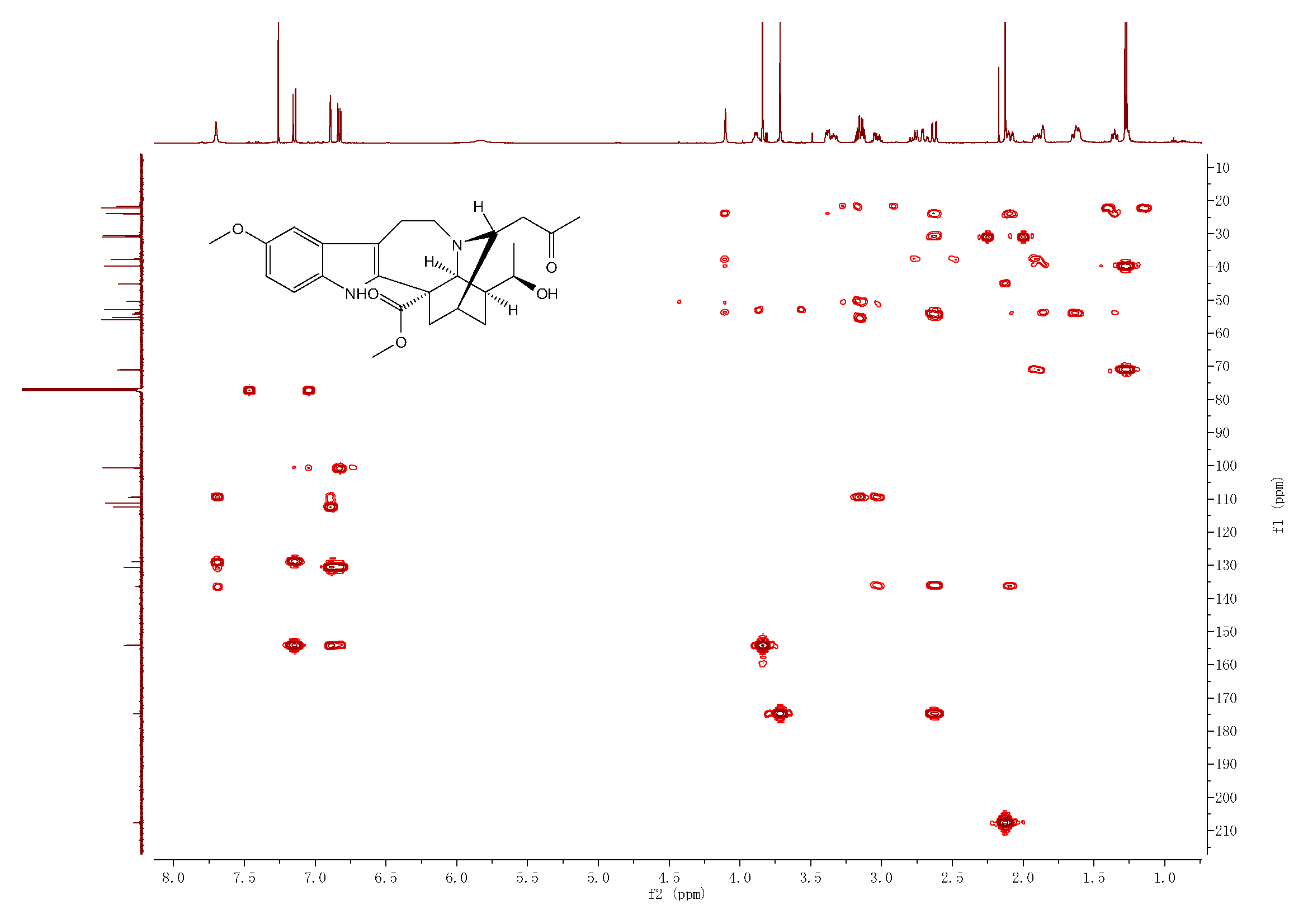


**S13.** ROESY spectrum (500 MHz, CDCl_3_) of (3*R*,19*R*)-19-hydroxy-3-(2-oxopropyl)voacangine (**2**)


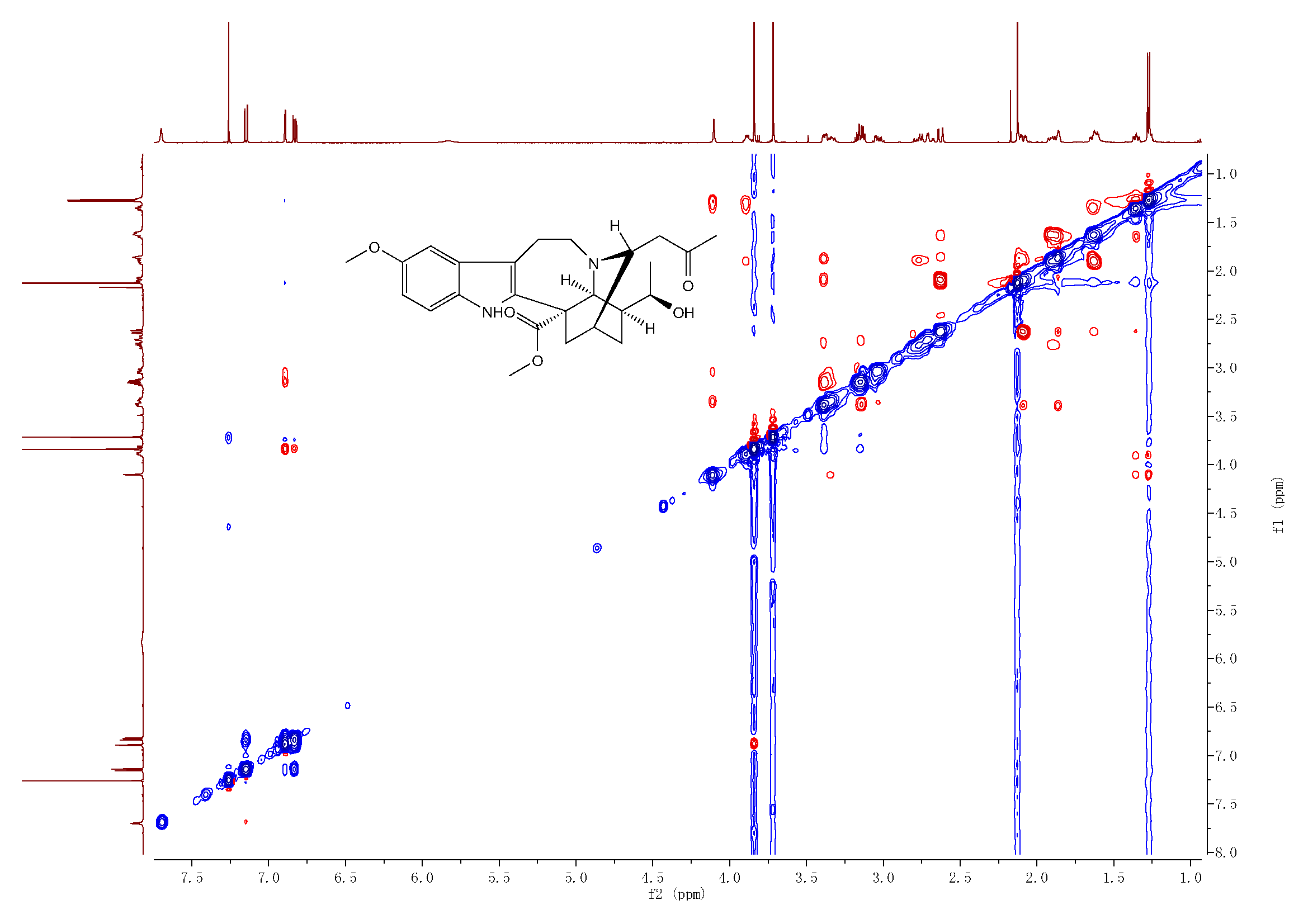


**S14**. CD spectrum of (3*R*,19*R*)-19-hydroxy-3-(2-oxopropyl)voacangine (**2**).


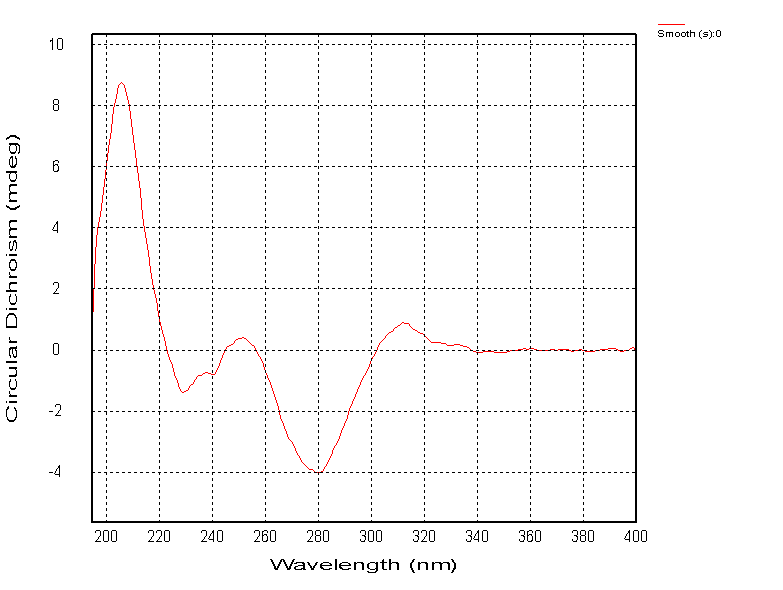

Supplement: Supplementary file 1 — Supplementary file1 (DOCX 786 kb) [file 13659_2019_226_MOESM1_ESM.docx]
